# Demographic, practice and clinical management characteristics of osteopaths referring to podiatrists: secondary analysis of a nationally representative sample of Australian osteopaths

**DOI:** 10.1186/s12913-022-07520-6

**Published:** 2022-02-18

**Authors:** Michael Fleischmann, Brett Vaughan, Adam Bird, Sandra Grace, Kylie Fitzgerald, Gopi McLeod

**Affiliations:** 1 College of Health and Biomedicine, Victoria University, Melbourne, Australia; 2 School of Public Health, University of Technology Sydney, Sydney, Australia; 3 Department of Medical Education, University of Melbourne, Melbourne, Australia; 4 School of Health and Human Sciences, Southern Cross University, Lismore, Australia; 5 School of Allied Health, Human Services and Sport, La Trobe University, Melbourne, Australia

## Abstract

**Background:**

Interprofessional care is paramount in contemporary healthcare practice. How different professions interact, and the characteristics of those practitioners who practice in an interprofessional way are rarely described in the literature. The aim of the current work was to identify the demographic, practice and clinical management characteristics of Australian osteopaths who report referring to podiatrists.

**Methods:**

The study was a secondary analysis of data from the Osteopathy Research and Innovation Network (ORION). Inferential statistics were generated to identify statistically significant demographic, practice and clinical management characteristics associated with referrals to podiatrists by Australian osteopaths.

**Results:**

Nine-hundred and ninety-two Australian osteopaths responded to the questionnaire. Sending referrals to a podiatrist was reported by 651 participants (65.6%). Female Australian osteopaths were less likely to report referring to podiatrists compared to male osteopaths (*OR* 0.76, 95%CI 0.59–0.99). Australian osteopaths who reported referring to podiatrists were more likely to report receiving referrals from podiatrists (*OR* 9.75, 95%CI 6.98–13.61), use orthopaedic testing in patient assessment (*OR* 7.62, 95%CI 2.82–20.60), and often treat patients with postural disorders (*OR* 1.71, 95%CI 1.03–2.26), compared to osteopaths who do not refer to podiatrists.

**Conclusion:**

This study provides initial evidence for the referral relationship between Australian osteopaths and podiatrists. Further work could explore the nature of these referrals, including the complaints resulting in referral and outcomes of care. This information will be useful to those involved in health policy development and the professions advocating for their role in the wider healthcare system.

## Background

Multidisciplinary care has been associated with benefits for patients and health professionals alike. These benefits include improved health outcomes, patient satisfaction, efficient use of resources, and job satisfaction for team members [1]. Working in a multidisciplinary care environment can be significantly influenced by context [2], particularly practice location and co-location with other health professionals [3, 4]. Health professionals located in tertiary care environments can readily engage in multidisciplinary care, given the ease of access to other health professionals. In the primary care environment, however, this immediacy of access may be more challenging to achieve and may require different pathways for patients to involve other health professionals in their care.

Australian podiatrists work in both public and private health contexts where they provide care for a range of lower limb conditions. In contrast, Australian osteopaths are predominantly located in private health care settings, where they provide care for a range of musculoskeletal complaints, including those affecting the lower limb [5–7]. Both professions are government registered in Australia, with accredited pre-registration programmes [8, 9]. At the end of 2019, there were 5509 registered podiatrists, with nearly 60% identifying as female [10], and 2723 registered osteopaths, with 55% identifying as female [11]. At the time over half of each the practitioners in each profession were less than 40 years of age. It is not possible to ascertain the practice location (public versus private) from this registration data; however, other works suggest that in Australia 90% of osteopaths [5] and approximately 72% of podiatrists [12] are located in private practice.

Australian podiatrists and osteopaths share several practice commonalities - in particular, the care of lower limb musculoskeletal complaints [5–7]. In Australia, services provided by both professions are included in the Medicare Chronic Disease Management (CDM) plan scheme [13], whereby patients can access, under Medicare, up to five consultations with allied health professionals (e.g. podiatrist, dietician, physiotherapist) per year to assist with the management of a chronic complaint, including musculoskeletal issues. This scheme entitles patients to a rebate from the Australian Government to assist with the costs of their care [13]. The CDM scheme presents an opportunity for osteopaths and podiatrists to work as part of a patient’s multidisciplinary care team. Menz [14] reported that in the 2004–2008 period over 1.3 million consultations for podiatry care and 82,486 consultations with osteopaths were funded over the same period through the CDM scheme [15], with the rebates facilitated through the Medicare EasyClaim system. The almost ten-fold difference between the number of podiatry consultations and the number of osteopathy consultations through the CDM scheme is likely to be due to podiatrists having a narrower specialisation (foot care), and hence less competition compared with osteopaths, who are just one type of provider of musculoskeletal care under the CDM scheme.

Practice-based research networks (PBRNs) foster research, develop practice-relevant research questions, and assist knowledge translation to improve clinical care [16, 17]. PBRNs have been used both in Australia and internationally across the medical and allied health professions [5, 16, 18–21]. The current study explored the demographic, practice and clinical-management characteristics of the practice of Australian osteopaths who send referrals to, and receive referrals from, podiatrists through the Osteopathy Research and Innovation Network (ORION) – the largest voluntary nationally representative PBRN in osteopathy worldwide [5, 22]. Little is known about the patterns of patient referrals between the osteopathy and podiatry professions (amongst other health professions) in Australia. An emerging picture of referrals for both of these professions in Australia suggests that referrals are made to and from a range of health professionals [5, 6, 23]. However, the characteristics of Australian osteopaths who send referrals to other health professionals has not been explored to date. The data from this secondary analysis of the ORION PBRN will not only contribute to our understanding of how these two professions work together in the Australian healthcare system but also inform interprofessional education in pre- and post-registration training programmes and assist with the development of health policy for interprofessional care.

## Method

### Sample

Nine-hundred and ninety-two (*N* = 992) responses were received, representing 49% of the osteopathy profession at time of completion in July to December 2016. Participants were required to be registered osteopaths practising in Australia. Study participants were recruited through the Australian osteopathy professional association (Osteopathy Australia) and by word of mouth. The baseline sample of participants (including that the sample is nationally representative on a number of key indicators) is extensively described elsewhere [5].

### Questionnaire

A questionnaire was developed to establish ORION baseline data and this data is described elsewhere [5, 22]. Participants were invited to complete a self-report 27-item questionnaire designed to collect a range of demographic (e.g., age, gender, highest osteopathy degree, other degrees, length of time in clinical practice), clinical (e.g., presenting conditions, patient groups treated, techniques and adjuncts applied) and practice characteristics (e.g., patient care hours per week, visits per week, co-located with other health professionals, referral to and/or from other health professionals). The questionnaire did not explore other individual practitioner characteristics such as ethnicity or income level. The analysis presented in this paper focuses on the demographic, practice and clinical management characteristics of osteopaths associated with referrals to Australian podiatrists.

### Statistical analyses

Participant responses were analysed based on whether the respondent reported sending patient referrals to podiatrists (outcome variable). Other variables were analysed in relation the independent variables, using independent t-tests for continuous variables and chi-square tests for categorical variables. Effect size and unadjusted odds ratios were calculated for statistically significant variables. Alpha was set at 0.05. All descriptive statistical analyses, t-tests, and chi-square tests were performed using SPSS version 25 (IBM Corp., Armonk, NY, USA).

## Results

Responses were received from all 992 osteopaths who responded to the ORION questionnaire question regarding sending referrals to a podiatrist (yes/no), with no missing data. One hundred and forty-seven respondents (*n* = 147, 14.8%) reported being co-located with a podiatrist. Sending referrals to a podiatrist was reported by 651 participants (65.6%) and receiving referrals from a podiatrist by 471 participants (47.5%). The demographic characteristics of Australian osteopaths who reported sending referrals are provided in Table 1. Female osteopaths were significantly less likely than male osteopaths to send referrals to podiatrists (*OR* 0.76, 95%CI 0.59–0.99), and those osteopaths who reported referring patients to podiatrists were likely to be younger with a small effect size (*p* < 0.01, d = 0.17).

**Table 1:** Comparison of practitioner characteristics of Australian osteopaths who report sending referrals to podiatrists with those who do not report referring

	Yes (***n*** = 651)	No (***n*** = 341)	***p***-value	Odds ratio [OR]*
**Gender**				
Male	393 (60.4%)	183 (53.7%)		
Female	258 (39.6%)	158 (46.3%)	0.04	0.76 [0.59, 0.99]
**Age (years)**				
Mean (±SD)	37.4 (±10.1)	39.2 (±12.0)	0.01^a^	
**Years in clinical practice**				
Mean (±SD)	11.4 (±8.3)	11.4 (±10.2)	0.99	
**Patient care hours per week**				
Mean (±SD)	28.3 (±12.1)	27.3 (±12.2)	0.25	
**Patient visits per week**				
Mean (±SD)	37.3 (±18.9)	35.0 (±18.0)	0.10	
**Qualification (n, %)^**				
Diploma	36 (5.5%)	26 (7.6%)	0.34	
Advanced diploma	5 (0.8%)	4 (1.2%)		
Bachelor degree	144 (22.1%)	74 (21.7%)		
Master’s degree	450 (69.1%)	231 (67.7%)		
PhD	2 (0.3%)	3 (0.9%)		
Other	14 (2.2%)	3 (0.9%)		
**Involved in as an osteopath**				
University teaching^#^	73 (11.2%)	43 (12.6%)	0.52	
Clinical supervision^#^	104 (16.0%)	46 (13.5%)	0.30	
Professional organisations	65 (10.0%)	42 (12.3%)	0.26	
Research	35 (5.4%)	19 (5.6%)	0.89	
Volunteer	110 (16.9%)	49 (1.4%)	0.30	

^*^ unadjusted odds ratio, ^ analysed as a categorical variable, ^#^ participation in the previous 12 months (yes/no), ^a^ d = 0.17 [0.04–0.30]

Osteopaths who reported being co-located with a podiatrist, were more likely to report sending referrals to a podiatrist (*OR* 3.08, 95%CI 1.94–4.87) or exercise physiologist (*OR* 2.27, 95%CI 1.43–3.59), compared to colleagues who did not report referring to podiatrists (Table 2). Australian osteopaths who reported sending referrals to podiatrists were also over four times more likely to send referrals to general practitioners (*OR* 4.62, 95%CI 3.05–7.01) and more than twice as likely to send referrals to a range of medical, allied health and complementary medicine professionals (Table 2). Osteopaths who reported sending referrals to podiatrists over nine times more likely to receive report receiving referrals from a podiatrist, compared to osteopaths who did not report referring (*OR* 9.75, 95%CI 6.98–13.61). Orthopaedic testing was more likely to be used by osteopaths who reported sending patient referrals to podiatrists (*OR* 7.62, 95%CI 8.82–20.60) and these osteopaths were 40% more likely to use the Medicare EasyClaim system to claim rebates under the CDM scheme [13] (Table 2).

**Table 2:** Comparison of practice characteristics of Australian osteopaths who report sending referrals to podiatrists with those who do not report referring

	Yes (***n*** = 651)	No (***n*** = 341)	***p***-value	Odds Ratio (OR)^**a**^ [95%CI]
**Practice location**				
Urban practice	538 (82.6%)	282 (82.7%)	0.98	–
More than one practice location	216 (33.2%)	131 (38.4%)	0.10	–
**Co-located with other health professionals (‘yes’)**				
Osteopath	432 (66.4%)	211 (61.9%)	0.16	–
General Practitioner	45 (6.9%)	27 (7.9%)	0.56	–
Specialist Medical Practitioner	15 (2.3%)	16 (4.7%)	0.04	0.48 [0.23, 0.98]
Podiatrist	123 (18.9%)	24 (7.0%)	< 0.01	3.08 [1.94, 4.87]
Physiotherapist	100 (15.4%)	44 (12.9%)	0.30	–
Exercise Physiologist	99 (15.2%)	25 (7.3%)	< 0.01	2.27 [1.43, 3.59]
Occupational Therapist	13 (2.0%)	6 (1.8%)	0.79	–
Psychologist	120 (18.4%)	71 (20.8%)	0.36	–
Massage Therapist	343 (52.7%)	158 (46.3%)	0.06	–
Acupuncturist	116 (17.8%)	72 (21.1%)	0.21	–
Naturopath	130 (20.0%)	63 (18.5%)	0.57	–
Dietician	49 (7.5%)	23 (6.7%)	0.65	–
Nutritionist	50 (7.7%)	28 (8.2%)	0.77	–
**Send referrals to other health professionals (‘yes’)**				
Osteopath	356 (54.7%)	150 (44.0%)	< 0.01	1.54 [1.18, 2.00]
General Practitioner	613 (94.2%)	265 (77.7%)	< 0.01	4.62 [3.05, 7.01]
Specialist Medical Practitioner	343 (52.7%)	100 (29.3%)	< 0.01	2.68 [2.03, 3.55]
Podiatrist	–	–	–	–
Physiotherapist	266 (40.9%)	65 (19.1%)	< 0.01	2.93 [2.15, 4.00]
Exercise Physiologist	306 (47.0%)	92 (27.0%)	< 0.01	2.40 [1.80, 3.19]
Occupational Therapist	87 (13.4%)	19 (5.6%)	< 0.01	2.61 [1.56, 4.37]
Psychologist	265 (40.7%)	84 (24.6%)	< 0.01	2.10 [1.57, 2.81]
Massage Therapist	492 (75.6%)	179 (52.5%)	< 0.01	2.80 [2.12, 3.70]
Acupuncturist	335 (51.5%)	116 (34.0%)	< 0.01	2.05 [1.56, 2.70]
Naturopath	356 (54.7%)	121 (35.5%)	< 0.01	2.19 [1.67, 2.87]
Dietician	136 (20.9%)	31 (9.1%)	< 0.01	2.64 [1.74, 3.99]
Nutritionist	94 (14.4%)	35 (10.3%)	0.06	–
**Receive referrals from other health professionals (‘yes’)**				
Osteopath	429 (65.9%)	185 (54.3%)	< 0.01	1.63 [1.24, 2.13]
General Practitioner	594 (91.2%)	292 (85.6%)	< 0.01	1.75 [1.16, 2.62]
Specialist Medical Practitioner	168 (25.8%)	69 (20.2%)	0.05	–
Podiatrist	418 (64.2%)	53 (15.5%)	< 0.01	9.75 [6.98, 13.61]
Physiotherapist	190 (29.2%)	76 (22.3%)	0.02	1.44 [1.06, 1.95]
Exercise Physiologist	196 (30.1%)	62 (18.2%)	< 0.01	1.94 [1.40, 2.67]
Occupational Therapist	47 (7.2%)	14 (4.1%)	0.05	–
Psychologist	96 (14.7%)	58 (17.0%)	0.35	–
Massage Therapist	529 (81.3%)	225 (66.0%)	< 0.01	2.23 [1.66, 3.01]
Acupuncturist	265 (40.7%)	105 (30.8%)	< 0.01	1.54 [1.17, 2.04]
Naturopath	285 (43.8%)	115 (33.7%)	< 0.01	1.53 [1.16, 2.01]
Dietician	28 (4.3%)	11 (3.2%)	0.41	–
Nutritionist	37 (5.7%)	18 (5.3%)	0.79	–
**Diagnostic imaging**				
Referral for imaging (‘often’)	48 (7.4%)	25 (7.3%)	0.98	–
Investigation of unknown pathologies	496 (76.2%)	246 (72.1%)	0.16	–
Investigation of suspected diagnosis	562 (86.3%)	273 (80.1%)	0.01	1.57 [1.11, 2.22]
Investigation of potential fractures	496 (76.2%)	254 (74.5%)	0.55	–
Rule out risk factors prior to treatment	174 (26.7%)	98 (28.7%)	0.50	–
General screening of the spine	13 (2.0%)	19 (5.6%)	< 0.01	0.34 [0.17, 0.71]
**Patient assessment (‘yes’)**				
Orthopaedic testing	646 (99.2%)	322 (94.4%)	< 0.01	7.62 [2.82, 20.60]
Clinical assessment algorithm	320 (49.2%)	148 (43.4%)	0.08	–
Neurological testing	611 (93.9%)	307 (90.0%)	0.03	1.69 [1.05, 2.72]
Screening questionnaire	433 (66.5%)	200 (58.7%)	0.01	1.40 [1.07, 1.83]
Cranial nerve testing	443 (68.0%)	229 (6.72%)	0.77	–
**Payment strategies**				
HICAPS	603 (92.8%)	301 (88.8%)	0.03	1.62 [1.03, 2.54]
Medicare EasyClaim	302 (46.4%)	129 (37.8%)	0.01	1.42 [1.09, 1.86]

^a^ unadjusted odds ratio

Australian osteopaths who reported sending referrals to podiatrists were more likely to report often treating low back pain (*OR* 3.11, 95%CI 1.00–9.57), postural disorders (*OR* 1.71, 95%CI 1.03–2.26) and tendinopathies (*OR* 1.38, 95%CI 1.06–1.81), than their counterparts who did not send patient referrals to a podiatrist (Table 3).

**Table 3:** Comparison of clinical management characteristics of Australian osteopaths who report sending referrals to podiatrists with those who do not report referring

	Yes (***n*** = 651)	No (***n*** = 341)	***p***-value	Odds Ratio (OR)* [95%CI]
**Discuss with patients (‘often’)**				
Diet	239 (36.8%)	136 (39.9%)	0.34	–
Smoking and drug use	115 (1.7%)	64 (18.8%)	0.68	–
Physical activity	591 (90.9%)	295 (86.5%)	0.03	1.56 [1.04, 2.35]
Occupation Health & Safety	346 (53.4%)	160 (46.9%)	0.05	–
Pain counselling	175 (26.9%)	91 (26.7%)	0.95	–
Stress	314 (48.4%)	175 (51.5%)	0.35	–
Nutrition	158 (24.3%)	94 (27.6%)	0.26	–
Medication	262 (40.3%)	129 (37.9%)	0.47	–
**Patient presentations (‘often’)**				
Neck pain	642 (98.8%)	329 (96.5%)	0.01	2.93 [1.19, 7.23]
Thoracic pain	600 (92.3%)	309 (90.6%)	0.36	–
Low back pain	645 (99.2%)	332 (97.6%)	0.04	3.11 [1.00, 9.57]
Hip musculoskeletal pain	500 (76.9%)	244 (71.8%)	0.08	–
Knee musculoskeletal pain	324 (50.0%)	167 (49.1%)	0.79	–
Ankle musculoskeletal pain	226 (34.9%)	107 (31.4%)	0.27	–
Foot musculoskeletal pain	197 (30.4%)	97 (28.4%)	0.53	–
Shoulder musculoskeletal pain	539 (83.1%)	262 (77.1%)	0.02	1.46 [1.05, 2.02]
Elbow musculoskeletal pain	164 (25.4%)	87 (25.6%)	0.94	–
Wrist musculoskeletal pain	122 (18.8%)	66 (19.4%)	0.84	–
Hand musculoskeletal pain	74 (11.5%)	47 (13.9%)	0.27	–
Postural disorders	469 (72.4%)	206 (60.4%)	< 0.01	1.71 [1.03, 2.26]
Degenerative spine conditions	411 (63.4%)	188 (55.1%)	0.01	1.41 [1.08, 1.84]
Headache disorders	673 (68.0%)	219 (22.1%)	0.43	–
Migraine disorders	264 (40.7%)	136 (40.0%)	0.82	–
Spine health maintenance	303 (46.8%)	155 (45.5%)	0.68	–
Chronic or persistent pain	405 (62.5%)	225 (66.0%)	0.28	–
Tendinopathies	286 (44.1%)	124 (36.4%)	0.02	1.38 [1.06, 1.81]
Temporomandibular joint disorders	115 (17.7%)	68 (20.1%)	0.37	–
Non-musculoskeletal disorders	65 (10.1%)	61 (18.0%)	< 0.01	0.51 [0.35, 0.75]
**Patient subgroups (treat ‘often’)**				
Up to 3 years of age	96 (14.8%)	60 (17.6%)	0.24	–
4 to 18 years of age	186 (28.6%)	84 (24.6%)	0.18	–
Over 65 years of age	386 (59.4%)	186 (54.5%)	0.14	–
Aboriginal & Torres Strait Islander peoples	7 (1.1%)	0 (0%)	0.05	–
Pregnancy	237 (36.5%)	107 (31.4%)	0.11	–
Non-English speaking	24 (3.7%)	9 (2.6%)	0.38	–
Sport injuries	350 (53.8%)	151 (44.4%)	< 0.01	1.46 [1.12, 1.90]
Worker injury (compensable)	68 (10.5%)	35 (10.3%)	0.91	–
Work injury (non-compensable)	238 (36.6%)	103 (30.2%)	0.04	1.33 [1.01, 1.77]
Traffic injury (compensable)	36 (5.6%)	18 (5.3%)	0.85	–
Traffic injury (non-compensable)	73 (11.2%)	41 (12.1%)	0.68	–
Post-surgery	52 (8.0%)	27 (8.0%)	0.98	–
**Manual therapy (use ‘often’)**				
Counterstrain	293 (45.1%)	127 (37.2%)	0.01	1.38 [1.06, 1.81]
Muscle energy technique	546 (84.0%)	242 (71.0%)	< 0.01	2.15 [1.57, 2.94]
High-velocity, low-amplitude manipulation	441 (67.8%)	191 (56.0%)	< 0.01	1.65 [1.26, 2.17]
Joint manipulation	281 (43.4%)	112 (32.8%)	< 0.01	1.56 [1.19, 2.06]
Soft tissue technique	584 (90.0%)	264 (77.4%)	< 0.01	2.62 [1.83, 3.76]
Myofascial release	426 (65.6%)	186 (54.5%)	< 0.01	1.59 [1.22, 2.09]
Visceral techniques	56 (8.6%)	42 (12.3%)	0.06	–
Lymphatic pump	52 (8.0%)	32 (9.4%)	0.46	–
Autonomic balancing	93 (14.3%)	64 (18.8%)	0.06	–
Biodynamics	88 (13.5%)	67 (19.6%)	0.01	–
Functional technique	176 (27.1%)	94 (27.6%)	0.87	–
Balanced ligamentous tension	216 (33.2%)	133 (39.0%)	0.07	–
Chapman’s reflexes	16 (2.5%)	8 (2.4%)	0.91	–
Trigger point therapy	199 (30.6%)	59 (17.4%)	< 0.01	–
Osteopathy in the Cranial Field	141 (21.7%)	92 (27.0%)	0.06	–
Facilitated positional release	115 (17.7%)	51 (15.0%)	0.29	–
Dry needling	170 (26.2%)	64 (18.8%)	0.01	1.52 [1.10, 2.11]
Exercise prescription	500 (77.0%)	233 (68.3%)	< 0.01	1.55 [1.16, 2.08]
Shockwave therapy	11 (1.7%)	7 (2.1%)	0.68	–
Ultrasound	14 (2.2%)	13 (3.8%)	0.13	–
Transcutaneous Electrical Nerve Stimulation (TENS)	7 (1.1%)	12 (3.5%)	< 0.01	0.29 [0.11, 0.76]
Instrument manipulation	0 (0%)	2 (0.6%)	0.05	–
Instrument soft tissue	8 (1.2%)	4 (1.2%)	0.94	–
Sport taping	93 (14.3%)	29 (8.5%)	< 0.01	1.79 [1.15, 2.79]
**Expanded practice scope (‘definitely’)**				
Prescribing rights	175 (26.9%)	82 (24.0%)	0.32	–
Referral rights to orthopaedic surgeon	475 (73.1%)	228 (66.9%)	0.04	1.34 [1.01, 1.79]
Referral rights to paediatrician	350 (53.8%)	190 (55.7%)	0.57	–
Referral rights to sports medicine specialist	542 (83.5%)	248 (72.7%)	< 0.01	1.90 [1.39, 2.60]
Referral rights to rheumatologist	424 (65.2%)	205 (60.1%)	0.11	–
Referral rights to other medical specialist	1 (0.2%)	0 (0%)	0.46	–
Expanded diagnostic imaging rights	551 (84.8%)	271 (79.5%)	0.03	1.44 [1.02, 2.02]
**Research (‘strongly agree’)**				
Help patients understand osteopathy	284 (43.6%)	159 (46.6%)	0.36	–
Help general practitioners and other health professionals understand osteopathy	441 (70.3%)	229 (70.5%)	0.96	–
Provide scientific evidence	324 (52.4%)	191 (59.5%)	0.04	0.75 [0.57, 0.98]
Irrelevant to the development of osteopathy^a^	348 (56.4%)	215 (67.2%)	< 0.01	0.63 [0.47, 0.84]

^*^ unadjusted odds ratio, ^a^ ‘strongly disagree’

## Discussion

The current study identified the demographic, practice and clinical management characteristics of Australian osteopaths who report sending referrals to podiatrists, compared to their colleagues who do not report referring. A significant observation was the number of Australian osteopaths reporting sending referrals to podiatrists - approximately two-thirds reported sending referrals to podiatrists [5]. This observation, alongside the large association with receiving referrals from a podiatrist, suggests that a strong referral relationship exists between Australian osteopaths and osteopaths are utilising the expertise of podiatrists for patient care. These observations also potentially reflect a shared understanding between osteopaths and podiatrists of the role that each profession plays in patient care. Data from some studies indicate instances of multidisciplinary care involving podiatrists as part of the team [24–26]. These studies describe podiatrists positively contributing to the multidisciplinary care team for arthritic and ulcerative conditions; however, there is little literature describing multidisciplinary care involving osteopaths [27] and none in Australia.

Through secondary analysis of data from a national osteopathy PBRN, it appears that Australian osteopaths who report sending referrals to podiatrists are likely to engage in referrals with other allied health and complementary medicine professions. This finding is encouraging given the need for interprofessional patient care of musculoskeletal complaints [28], and has rarely been demonstrated in the literature describing the Australian osteopathy profession. The data presented here may be useful for informing health policy development around multidisciplinary care for musculoskeletal complaints (i.e. how best to utilise available health resources for patient care), and in patient care for chronic conditions given their significant cost to the healthcare system [29]. There is also an opportunity to use the data in the current study to inform the pre-registration education of Australian osteopaths and podiatrists, particularly focusing on interprofessional care.

Regarding practitioner characteristics, the current study identified two significant variables. The first was that female osteopaths were approximately 30% less likely to send referrals to podiatrists compared to their male counterparts in the unadjusted modelling. The reason for this difference in referrals based on gender requires additional exploration. Secondly, younger osteopaths were also more likely to report sending referrals to podiatrists compared to older colleagues. Whether this reflects an increase in knowledge of the role of podiatrists in musculoskeletal complaint care through pre-professional education, or experience from practice (or both) would need additional research. However, the finding is encouraging from the multidisciplinary care perspective.

Australian osteopaths who reported sending referrals to podiatrists were over seven times more likely to utilise orthopaedic testing in patient assessment, compared to osteopaths who did not report referring. Orthopaedic testing is based on stressing musculoskeletal tissues and may be utilised to assist in developing a working diagnosis for the patient’s complaint. Further, these tests are utilised by both podiatrists [30] and osteopaths [31]. It may be that this shared understanding of the orthopaedic tests relevant to the respective professions practice may be being captured in the current secondary analysis. Again, such an assertion would benefit from additional research to explore this potential shared understanding of musculoskeletal examination procedures.

From a clinical management perspective, Australian osteopaths who send referrals to podiatrists were over 70% more likely to report treating postural disorders. Studies have described the relationship between the prescription of foot orthoses and changes in posture in both healthy [32, 33] and clinical populations [34]. Although a direct relationship cannot be established from the current dataset, it may be that postural disorders are a commonly referred issue for shared management of patients given they are reported to be managed by over one-third of Australian osteopaths [7]. Whether postural disorders are a key driver of referrals would be an interesting avenue for further research. It would be valuable to understand the clinical reasoning of osteopaths and podiatrists in the management of postural disorders. Additional research could also be directed towards understanding other conditions that may result in frequent referrals between the two professions.

Australian osteopaths who reported sending referrals to podiatrists were less likely, than their non-referring colleagues, to report treating non-musculoskeletal conditions. The dominant practice of Australian osteopaths relates to the management of musculoskeletal complaints [5, 6]. However, there is a subsection of the Australian osteopathic profession that apply manual therapy techniques to assist in the management of non-musculoskeletal complaints [5]. There is varying evidence to support the effectiveness of osteopathy care for non-musculoskeletal complaints [35, 36]. It may be that the nature of the non-musculoskeletal complaints managed by some Australian osteopaths does not require referral to a podiatrist. Rather, osteopaths appear to be referring to podiatrists for musculoskeletal complaints. The nature of the conditions resulting in referral to a podiatrist requires further investigation.

The bivariate analysis undertaken indicates the payment processes that might be associated with osteopathy and podiatry co-management of patients. Australian osteopaths who send referrals to podiatrists were over 40% more likely to use the Medicare EasyClaim service than osteopaths who do not send referrals. Both professions are eligible to treat patients under the CDM scheme, whereby patients with chronic diseases can access government funded care for up to five consultations with allied health professionals [13]. Medicare EasyClaim allows patients to claim the rebate at the time of the consultation upon referral from the patients’ general practitioner. It may be that patients under this CDM scheme are utilising both osteopathy and podiatry services, and the increased use of Medicare EasyClaim reflects this. This assertion may be supported by the large association observed in the current secondary analysis with osteopaths sending referrals to general practitioners. Additional investigations would assist in testing these assertions to understand how osteopathy and podiatry services are used under the CDM scheme.

The limitation of the current research is associated with the cross-sectional nature of the questionnaire. Questionnaires used with these study designs are potentially susceptible to social desirability and acquiescence biases [37], and the latter may have influenced responses to the send and receive referral items on the questionnaire. Further, it is not possible to comment on the frequency of referrals between the two professions given the dichotomous nature of the responses to these items.

## Conclusion

This study offers an initial empirical examination of the referral relationships between Australian osteopaths and podiatrists. Our secondary analysis shows Australian osteopaths who report sending referrals to podiatrists are also more likely to engage in referrals with other allied health and complementary medicine professions. The data presented here offers a resource for informing health policy development and has the potential for use in the pre-registration education of Australian osteopaths and podiatrists. Further research could develop a deeper understanding of the nature and frequency of the referrals between Australian osteopaths and podiatrists, including how these two professions work together through the Medicare CDM scheme for the benefit of patients.

## Data Availability

The datasets analysed during the current study are not publicly available as the authors do not have the authority to disseminate the data. The dataset is available through reasonable request at arccim@uts.edu.au.
